# Function and Regulation of *AUTS2*, a Gene Implicated in Autism and Human Evolution

**DOI:** 10.1371/journal.pgen.1003221

**Published:** 2013-01-17

**Authors:** Nir Oksenberg, Laurie Stevison, Jeffrey D. Wall, Nadav Ahituv

**Affiliations:** 1 Department of Bioengineering and Therapeutic Sciences, University of California San Francisco, San Francisco, California, United States of America; 2 Institute for Human Genetics, University of California San Francisco, San Francisco, California, United States of America; 3 Department of Epidemiology and Biostatistics, University of California San Francisco, San Francisco, California, United States of America; Yale University, United States of America

## Abstract

Nucleotide changes in the *AUTS2* locus, some of which affect only noncoding regions, are associated with autism and other neurological disorders, including attention deficit hyperactivity disorder, epilepsy, dyslexia, motor delay, language delay, visual impairment, microcephaly, and alcohol consumption. In addition, *AUTS2* contains the most significantly accelerated genomic region differentiating humans from Neanderthals, which is primarily composed of noncoding variants. However, the function and regulation of this gene remain largely unknown. To characterize *auts2* function, we knocked it down in zebrafish, leading to a smaller head size, neuronal reduction, and decreased mobility. To characterize *AUTS2* regulatory elements, we tested sequences for enhancer activity in zebrafish and mice. We identified 23 functional zebrafish enhancers, 10 of which were active in the brain. Our mouse enhancer assays characterized three mouse brain enhancers that overlap an ASD–associated deletion and four mouse enhancers that reside in regions implicated in human evolution, two of which are active in the brain. Combined, our results show that *AUTS2* is important for neurodevelopment and expose candidate enhancer sequences in which nucleotide variation could lead to neurological disease and human-specific traits.

## Author Summary

Autism spectrum disorders (ASDs) are neurodevelopmental disorders that affect 1 in 88 individuals in the United States. Many gene mutations have been associated with autism; however, they explain only a small part of the genetic cause for this disorder. One gene that has been linked to autism is *AUTS2*. *AUTS2* has been shown to be disrupted in more than 30 individuals with ASDs, both in coding and noncoding sequences (regions of the gene that do not encode for protein). However, its function remains largely unknown. We show here that *AUTS2* is important for neuronal development in zebrafish. In addition, we characterize potential *AUTS2* regulatory elements (DNA sequences that instruct genes as to where, when, and at what levels to turn on) that reside in noncoding regions that are mutated in ASD individuals. *AUTS2* was also shown to be implicated in human evolution, having several regions where its human sequence significantly changed when compared to Neanderthals and non-human primates. Here, we identified four mouse enhancers within these evolving regions, two of which are expressed in the brain.

## Introduction

**Figure 1:**
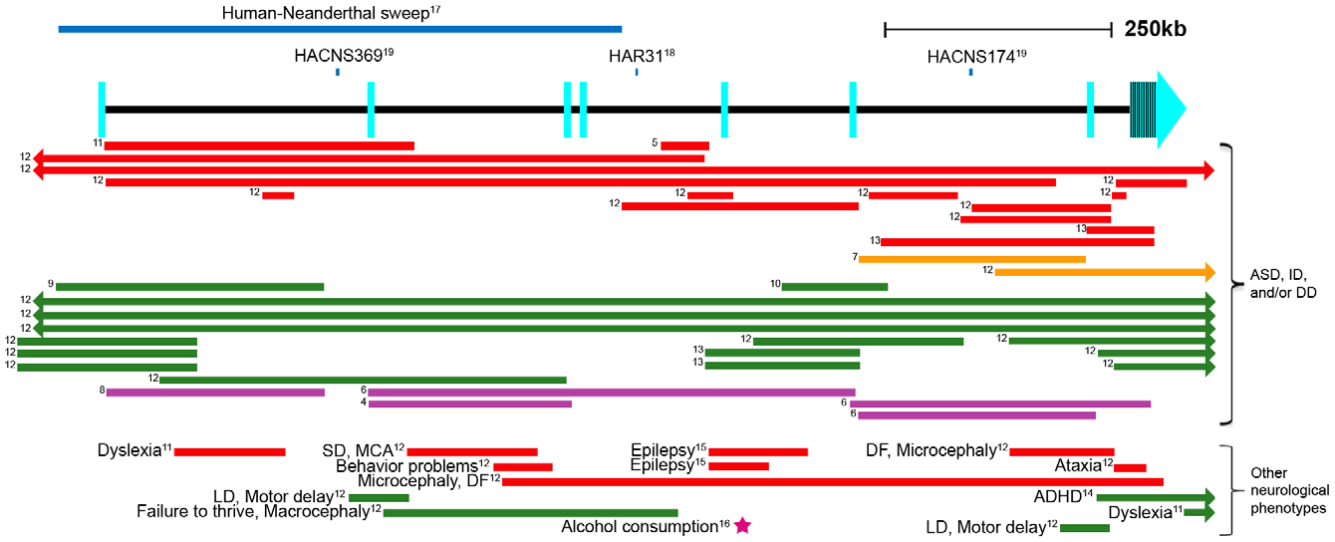
Schematic of the *AUTS2* genomic region. Human accelerated sequences are shown as blue lines above the gene [17]–[19]. Structural variants [4]–[12], [14], [15] are represented as colored lines (red: deletion, orange: inversion, green: duplication, purple: translocation). The rs6943555 SNP associated with alcohol consumption [16] is shown as a magenta star. Arrows in bars signify that the structural variant extends past the gene in that direction. Exons are depicted as light blue rectangles, as defined by the RefSeq genes track in the UCSC Genome Browser [52]. Numbers to the left of the lines correspond to a reference number. Human Accelerated Conserved Non-coding Sequence (HACNS), Human Accelerated Region (HAR), developmental delay (DD), intellectual disability (ID), dysmorphic features (DF), seizure disorder (SD), multiple congenital anomalies (MCA), language disability (LD).

Autism spectrum disorders (ASDs) are common (1/88 in the United States) [1] childhood neurodevelopmental disorders known as pervasive developmental disorders (reviewed in [2]). ASDs are highly heritable, signifying a substantial genetic etiology [3]. A balanced translocation involving the *autism susceptibility candidate 2* (*AUTS2*; GenBank NM_001127231.1) gene in a pair of monozygotic twins with ASD was the first to link this gene to autism [4] (Figure 1). Following this finding, thirty-six additional unrelated individuals with ASD, intellectual disability, or developmental delay were found to have distinct heterozygous structural variants disrupting the *AUTS2* region [5]–[13], four exclusively in noncoding regions [5], [12] (Figure 1). Additional structural variants in *AUTS2*, some of which are only intronic, were also shown to be associated with attention deficit hyperactivity disorder (ADHD) [14], epilepsy [12], [15], dyslexia [11], motor delay, language delay, visual impairment, microcephaly and others [12]. In addition, a genome-wide association meta-analysis study identified SNP rs6943555 within the fourth intron of *AUTS2* to be the most statistically significant SNP associated with alcohol consumption [16] (Figure 1). These various *AUTS2*-associated phenotypes suggest this gene has an important neurological function. It is worth noting though that some individuals with disrupted *AUTS2* and mental retardation or autism have additional, potentially non-neuronal phenotypes, such as hypotonia, short stature, urogenital abnormalities, and skeletal abnormalities [4], [6].

In addition to *AUTS2's* role in neurological disease, it was also shown to be important for human-specific evolution. The first half of *AUTS2* displayed the strongest statistical signal in a genomic screen differentiating modern humans from Neanderthals [17]. This is attributed to a stretch of 293 consecutive SNPs, only two of which are coding variants: (a G to C nonsynonymous substitution at chr7:68,702,743 (hg18) only in the Han Chinese and a C to T synonymous change in chr7:68,702,866 (hg18) within the Yoruba and Melanesian populations). Other regions identified to have the most significant human-Neanderthal sweeps also include genes that are involved in cognition and social interaction, including *DYRK1A*, *NRG3* and *CADPS2* [17], reinforcing our interest in *AUTS2*'s role in cognition and human-Neanderthal differences. In addition, three different evolutionary conserved noncoding intronic regions in *AUTS2* (HAR31, HACNS174 and HACNS369) have been found to be significantly accelerated when compared to primates in two different studies [18], [19] (Figure 1). Combined, these data suggest that altered regulation of *AUTS2* could be associated with human specific traits.

The functional role of *AUTS2* is not well known, although some studies have identified a putative role in transcriptional regulation during neuronal development. The predicted AUTS2 protein contains a PY motif, a putative WW-domain-binding region [4] present in various transcription factors, implying that *AUTS2* may be involved in transcriptional regulation [6]. In humans, *AUTS2* is expressed in the brain, including the neocortex and prefrontal cortex [4], [20]. *AUTS2* is also highly expressed in the skeletal muscle and the kidney, and in lower levels in the placenta, lung and leukocytes [4]. In the developing mouse, *Auts2* is expressed in the forebrain, midbrain, hindbrain, olfactory bulb, olfactory epithelium, eye, neural tube and limb [21]. Among the regions that *Auts2* was shown to be expressed in the brain are the neuronal nuclei in the developing cerebral cortex and cerebellum [22]. In the cortical preplate, *Auts2* is activated by T-box brain 1 (*Tbr1*) [22], [23], a postmitotic projection neuron specific transcription factor that is critical for normal brain development. *Tbr1* deficient mice display irregular laminar organization of cortical neurons [24]. Additionally, Cajal-Retzius cells in *Tbr1* deficient mice have decreased levels of reelin (*Reln*) [23], a protein that is involved in neuronal migration in the developing brain and has been reported to be expressed at decreased levels in individuals with ASD [25].

In this study, we used zebrafish morpholinos to functionally characterize *auts2*. We show that knocking down this gene leads to an overall stunted developmental phenotype that includes a smaller head, body and reduced movement. Further characterization of morphant fish revealed a reduction in developing midbrain neurons and also in sensory and motor neurons. To characterize *AUTS2* enhancers, we used both zebrafish and mouse transgenic enhancer assays. We identified three functional enhancers within an ASD-associated deletion and six brain enhancer in regions associated with human specific evolution. Combined, we found that *AUTS2* is important for neuronal development and characterized several functional enhancers within this locus, where nucleotide changes could be associated with neurodevelopmental disease and human specific evolution.

## Results

### *auts2* zebrafish expression

Zebrafish can be an effective tool to study ASD [26]. Using whole mount *in situ* hybridization, we determined that *auts2* is expressed in zebrafish at 24 hours post fertilization (hpf) in the forebrain, midbrain and hindbrain (Figure S1A). Additionally, *auts2* is expressed in the trunk (including the spinal cord), with stronger expression towards the caudal peduncle. At 48 hpf, *auts2* is expressed in the brain and pectoral fin and from 72–120 hpf its expression is restricted primarily to the brain. *auts2* is also weakly expressed in the eye from 24–120 hpf. Overall, we observed that the zebrafish expression largely correlates with the previously characterized mouse expression [21], [22].

### Phenotypic characterization of *auts2* morphants

**Figure 2:**
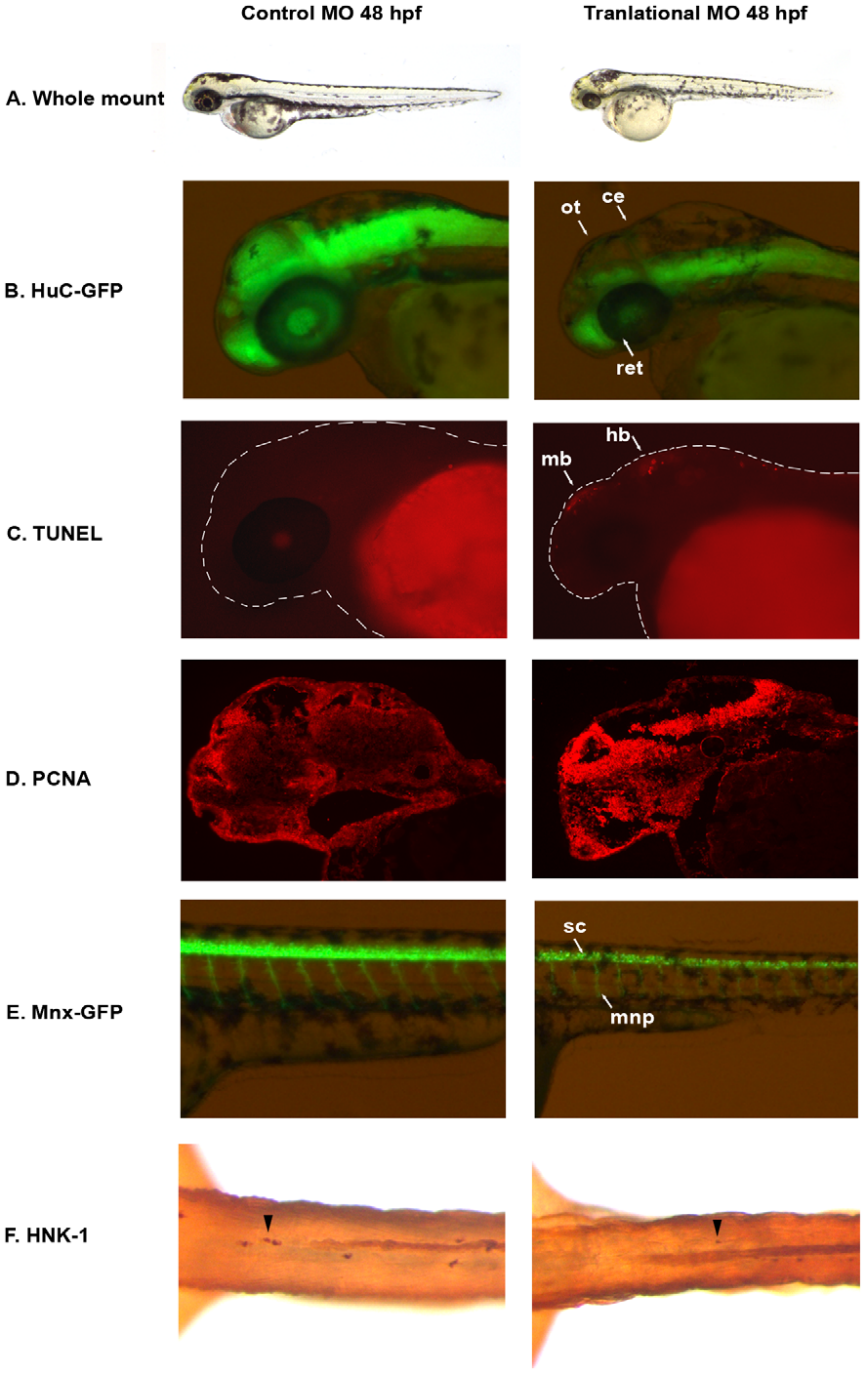
*auts2* 48 hpf morphant phenotype. (A) Fish injected with the 5 base-pair translational MO mismatch control have similar morphology as wild type fish. Injection of the *auts2* translational MO results in fish with a stunted development phenotype that includes a smaller head, eyes, body and fins. (B) HuC-GFP fish injected with the 5 bp control MO display normal levels of developing neurons in the brain. HuC-GFP translational MO injected fish display considerably less developing neurons in the optic tectum (ot), retina (ret), and cerebellum (ce). (C) 5 bp mismatch control injected fish have little to non-observable apoptosis in the brain as observed by TUNEL staining, while translational MO injected fish display high levels of apoptosis, primarily in the midbrain (mb) and hindbrain (hb). (D) PCNA cell proliferation assay in the 5 bp MO control injected fish shows lower levels of cell proliferation in the brain compared to the translational MO injected fish. (E) Tg(mnx1∶GFP) fish injected with the 5 bp MO control display normal levels of motor neurons versus the *auts2* translational MO injected fish which have fewer motor neurons in the spinal cord (sc). In addition, motor neuron projections (mnp) are weaker and more perpendicular to the spinal cord. (F) Translational MO injected fish display fewer Rohon-Beard cells (arrowheads) in the spinal cord than morphants. All morphant fish are scaled to their 5 bp control counterparts.

We next used morpholinos (MOs) to knockdown *auts2* in zebrafish during development. Fish injected with an *auts2* translational blocking MO displayed a stunted developmental phenotype with smaller heads, eyes, body and pectoral fins (Figure 2A and Figure S1C). A second *auts2* MO that disrupts the splice junction between intron two and exon three exhibited similar but less severe phenotypes (Figure S1D). These phenotypes appeared in 80–90% of injected fish and were rescued by co-injecting the full length human *AUTS2* mRNA along with the translational blocking MO (68% of injected fish showed a partial to full rescue) (Figure S1E). Injection of a 5 base pair (bp) mismatch *auts2* translational MO control did not show any phenotype (Figure 2A and Figure S1B), further validating the specificity of our MOs to effectively knockdown *auts2* in zebrafish.

To further characterize the neurological function of *auts2*, we injected the translational MO into the HuC-GFP transgenic zebrafish line [27], where developing neurons express green fluorescent protein (GFP). Compared to the 5 bp mismatch control, translational MO injected fish showed a dramatic decrease in GFP at 48 and 72 hpf in the dorsal region of the midbrain, including the optic tectum, the midbrain-hindbrain boundary (which includes the cerebellum), the hindbrain and the retina (Figure 2B). This phenotype was also observed by staining neurons with Nissl at 48 hpf (Figure S2A). TUNEL staining of 48 hpf embryos revealed that morphant fish exhibit increased apoptosis in the midbrain in the same location where fewer neurons where observed (Figure 2C and Figure S2B). Anti-proliferating Cell Nuclear Antigen (PCNA) staining showed increased amounts of cell proliferation in morphant fish in the forebrain, midbrain and hindbrain (Figure 2D and Figure S2C–S2E). While seemingly contradictory, increased amounts of both TUNEL and PCNA positive cells has been previously shown, as cell death and proliferation could be coupled [28], [29]. It is conceivable that the increased PCNA positive cells are the result of morphant cells failing to differentiate into mature neurons, as seen in the HuC-GFP line. These results suggest that *auts2* may be involved in the production and maintenance of neurons in the zebrafish brain.

Both the translational and splicing morphant fish also showed a decreased movement response when gently prodded with a pipette tip compared to controls that began at 48 hpf (Video S1 and Video S2). This phenotype was observed until 120 hours when the zebrafish were euthanized. In order to determine whether motor neuron defects could explain this phenotype, we injected the translational MOs into the Tg(mnx1∶GFP) zebrafish line, which expresses GFP in developing motor neurons [30]. At 48 hpf, morphant fish displayed fewer GFP labeled motor neuron cell bodies in the spinal cord. Additionally, motor neuron projections were weaker and perpendicular to the spinal cord, in contrast to the angled projections of the control injected fish (Figure 2E). This phenotype was also confirmed using the znp-1 antibody to mark motor neuron axons [31] in control and morphant fish. Morphant fish consistently showed more branching of axons compared to controls (Figure S3). To assess sensory neuron defects, Rohon-Beard neurons were stained with anti-HNK-1 in control and translational MO injected fish at 48 hpf. Morphant fish displayed on average 60% fewer sensory neurons in the spinal cord (Figure 2F). These results suggest that loss of *auts2* in zebrafish could lead to motor and sensory neuron defects, which may play a role in their reduced movement and decreased response to touch.

### *AUTS2* enhancer characterization

Due to the observations that noncoding regions in the *AUTS2* locus are associated with neurological phenotypes and human-specific evolution, we set out to identify enhancers in this locus. To focus our search, we limited our candidates to be between the first exon and fifth intron, due to this region encompassing the human-Neanderthal sweep (exon 1–4; chr7:68,662,946-69,274,862 (hg18)) [17] and several noncoding nucleotide changes that have been associated with neurological phenotypes [5], [11], [12], [16]. *AUTS2* enhancer candidate (AEC) sequences were selected based on evolutionary conservation, embryonic mouse forebrain and midbrain ChIP-seq datasets [32] and nucleotide variants that define the human-Neanderthal sweep [17] (see methods). We also tested the human accelerated region (HAR) in intron four, HAR31 [18], and the human accelerated conserved non-coding sequences (HACNS) in introns one and six, HACNS 369 and HACNS 174 respectively [19]. Using these criteria, 40 AECs were selected for zebrafish enhancer assays (Table S1). These human sequences were cloned into the E1b-GFP-Tol2 enhancer assay vector and injected into zebrafish [33]. Of the 40 candidates, 23 were found to be functional enhancers, 22 of which showed enhancer activity in locations that overlap *auts2* expression in zebrafish and 10 that were active in the brain (Table S1 and Figure S4).

**Figure 3:**
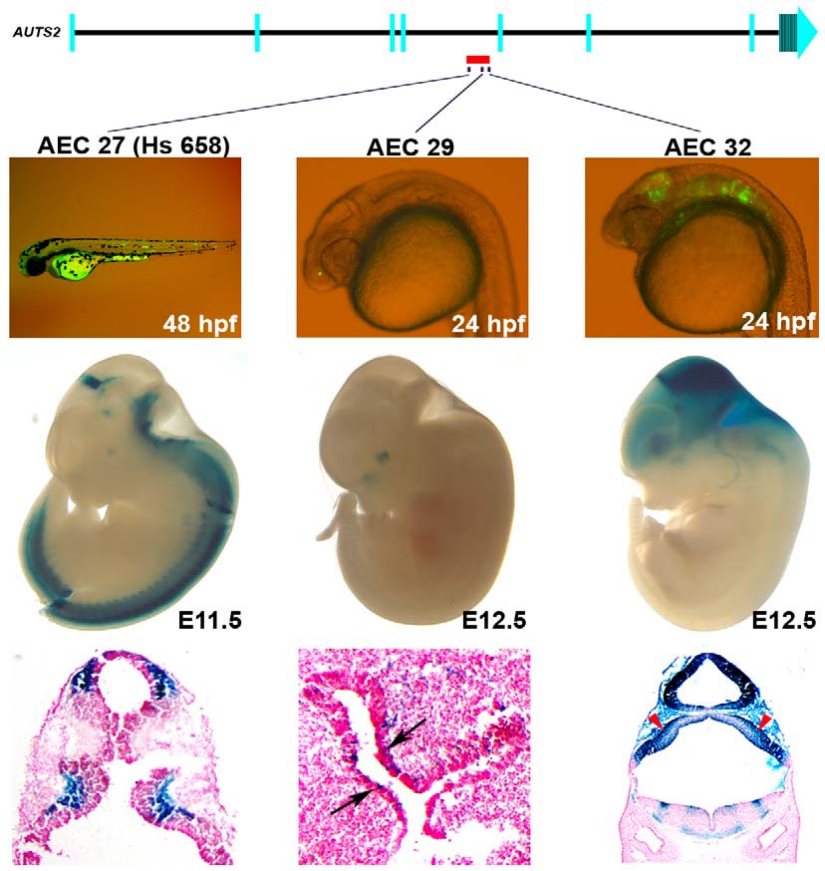
Enhancers within an ASD–associated *AUTS2* intronic deletion [**5**]. Three positive enhancers (AEC27, 29, 32) show positive enhancer activity in zebrafish (24 or 48 hpf) and in mice (E11.5 or 12.5). AEC27 shows enhancer expression in the somitic muscle in zebrafish, while in mouse at E11.5 (hs658; [34]) it is active in the midbrain, medulla, and neural tube at E11.5. The histological section below shows its enhancer activity in the pretectum and the pons. At E12.5, AEC29 shows enhancer activity in the olfactory epithelium (arrows in histological section) similar to zebrafish and in addition also displays enhancer expression in the eye. AEC32 recapitulates the zebrafish enhancer expression displaying strong enhancer activity in the midbrain (tectum) and hindbrain and in addition also displays enhancer expression in the forebrain at E12.5. Histological sections of AEC32 show enhancer activity in the mouse cerebellum (red arrowheads).

To further characterize the regulatory elements within a 33,519bp deletion associated with ASD in *AUTS2* intron four [5], the three positive zebrafish enhancers in this region (AEC27, AEC29, AEC32) were analyzed in mice using a similar transgenic assay [34]. AEC27 showed enhancer expression in the somitic muscle in zebrafish, while examination of its enhancer activity at E11.5 (hs658;[34]) found it to be active in the midbrain and neural tube (Figure 3). At E12.5, AEC29 had enhancer activity in the olfactory epithelium similar to zebrafish and also displayed enhancer expression in the eye (Figure 3). AEC32 recapitulated the zebrafish enhancer expression in the midbrain and hindbrain with additional enhancer expression in the forebrain at E12.5. Histological sections of AEC32 showed enhancer activity in the mouse cerebellum (Figure 3), a region thought to play a role in ASD [2]. The removal of these three brain enhancers and potentially other functional sequences in this region could contribute to the neurological phenotypes in patients with deletions in this intron.

**Figure 4:**
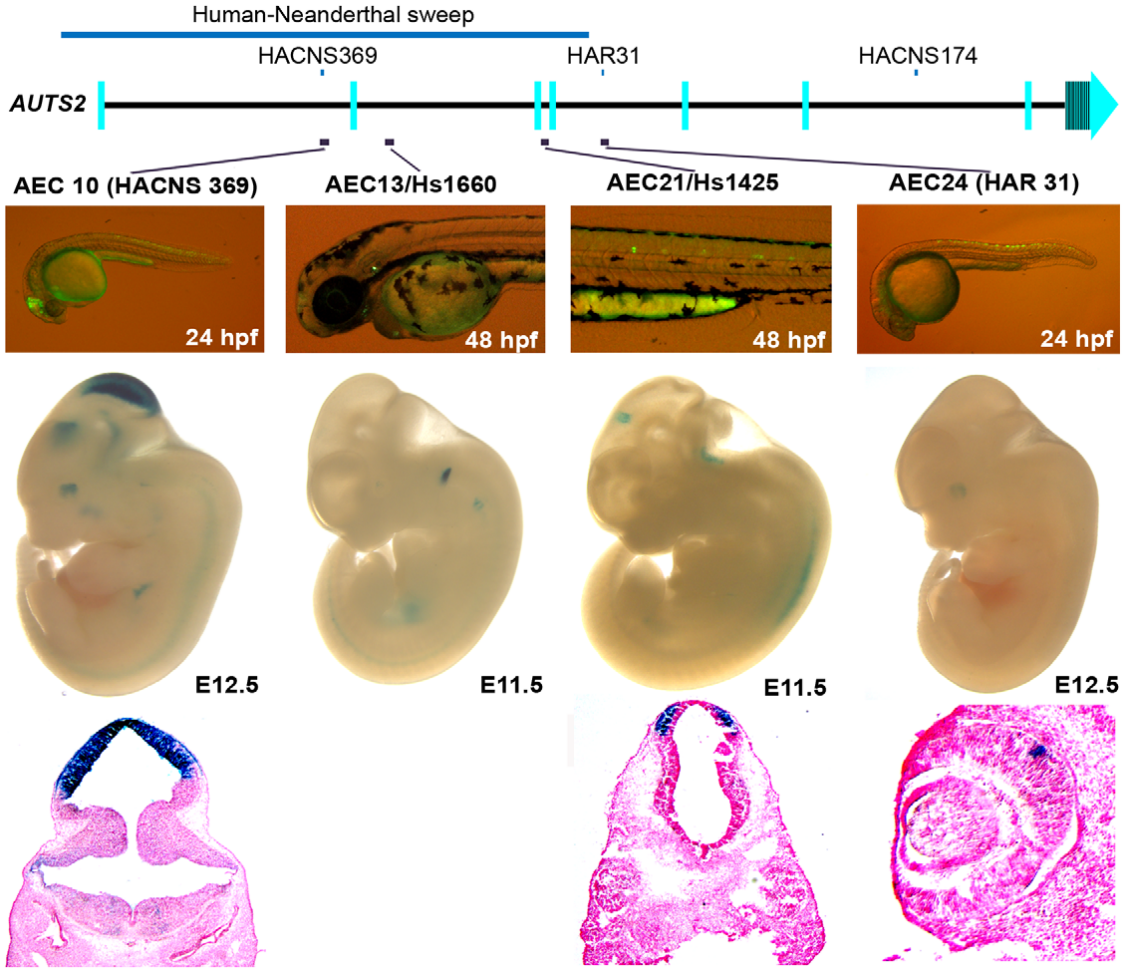
Four positive zebrafish and mouse enhancers in regions implicated in human evolution. At E12.5, AEC10 shows zebrafish and mouse enhancer expression in the midbrain and eye. The histological section below highlights its expression in the tectum. AEC13, is expressed in the otic vesicle both in zebrafish and E11.5 mouse embryos (hs1660 ; [34]). AEC21 is expressed in the spinal cord in zebrafish, while in the mouse it showed midbrain expression at E11.5 (hs1425; [34]). Histological sections below show its expression in the pretectum of the midbrain. AEC24 was expressed in the spinal cord and hindbrain in zebrafish and in the eye in mouse at E12.5.

We next set out to characterize enhancers in regions implicated in human-specific evolution. Four of the sixteen positive zebrafish enhancers identified in this region (Table S1 and Figure S4) were analyzed for enhancer activity in mice. These four sequences were positive mouse enhancers active in the brain, the otic vesicle, or eye (Figure 4 and Figure S5). Interestingly, two of these enhancers (AEC10 and 21) show enhancer expression in the developing tectum, a region in the brain that is thought to control auditory and visual responses.

## Discussion

Using MOs to knockdown *auts2*, we observed an overall phenotype of stunted development, making it difficult to characterize discrete phenotypes. However, using neuronal-labeled zebrafish lines and immunohistochemistry, we showed a reduction in motor and sensory neurons in the spinal cord and developing neurons in regions that include the midbrain and cerebellum. The cerebellum is involved in cognitive and emotional function and has been repeatedly implicated in ASD [2]. In addition, the cerebellum plays a major role in motor control, and it is possible that the defects detected in cerebellar neurons could partially explain the reduced movement phenotype observed in morphant fish. It is worth noting that two individuals with *AUTS2* structural variants had motor delay phenotypes (Figure 1) [12]. Given that the MO injected fish display additional phenotypes to the ones we focused on in this study, the effect of this gene on other tissues will need to be assessed in future experiments. Experiments such as mouse conditional knockouts should allow for a more complete understanding of *AUTS2* function. Our *auts2* MOs were designed to disrupt *auts2* activity on chromosome 10 (build Zv9). It is worth noting, that there is also a putative, less characterized version of *auts2* with an incomplete coding sequence located on zebrafish chromosome 15 (ENSDART00000012712). Knocking down this gene along with the *auts2* gene that was assayed in our study may lead to more severe phenotypes.

Our enhancer search focused primarily on the first five introns due to the numerous reports of cognitive-related structural variations in that region [4]–[6], [8]–, along with the region's putative role in evolution. There could be numerous functional enhancers outside this region that we have not tested in this study. For example, there is an intragenic SNP (rs6961611) associated with processing speed [35] 1.6 mega bases downstream of *AUTS2* which could be associated with a regulatory element for this gene. While the expression of our enhancers largely recapitulated *Auts2* expression, it is possible that the enhancers we identified could regulate a neighboring gene. Future experiments such as chromatin interaction analyses [36], [37] could be able to distinguish what promoters our enhancers are interacting with.

Previous work has shown that human enhancer sequences can function as active enhancers in zebrafish, even without homologous sequences in zebrafish [38]–[40]. Our results confirm these findings for some of our enhancers. For example, AEC10, 13 and 29, which do not have homologous sequences in zebrafish, have similar enhancer expression patterns in zebrafish and mouse (Table S1). However, AEC21 and 27, which are conserved down to zebrafish, and AEC 24, which is conserved down to chicken, don't have matching expression patterns in zebrafish and mice.

We found three positive human enhancers in both zebrafish and mouse that reside within a 33,519 bp deletion detected in an individual with ASD, one of which, AEC32, is expressed in the cerebellum. This deletion was inherited from the individual's mother who was not diagnosed with ASD [5]. ASDs are likely caused by multiple genomic aberrations in combination with environmental factors. While it is possible that in this individual, this deletion leads to ASD due to the loss of these enhancers and potentially other functional sequences, it is also possible that the loss of these enhancers is one of multiple “hits” [41] or that the deletion is not causative. With the constantly growing number of individuals with ASDs or other neurological phenotypes that have *AUTS2* mutations, some of which are purely noncoding, it is likely that improper regulation of this gene is involved in the progression of these disorders.

We also characterized enhancers in locations associated with other neurological phenotypes. In an 84 kb deletion in intron one of an individual with dyslexia, we identified four positive human enhancers in zebrafish (AEC3-6) (Table S1 and Figure S4), one of which is expressed in the midbrain. In addition, one of the candidates that was negative for zebrafish enhancer activity (AEC35) was a sequence that included the alcohol consumption associated SNP (rs6943555) [16]. It is possible that zebrafish is not a good model system for this region/phenotype or that the actual functional region/variant is further away from this tag SNP. By characterizing the regulatory landscape of this region we have obtained a better understanding of the functional units within this gene, which now pose as candidates for mutation analysis in individuals with various neurological phenotypes.

*AUTS2* has been singled out as a gene that is rapidly evolving in humans in three different studies [17]–[19]. Using zebrafish enhancer assays, we identified sixteen different enhancers that lie within regions that were implicated in human evolution, six of which show expression in the brain. We tested four of the enhancers in mice and two of them had midbrain enhancer activity. Our enhancer results, combined with the observation that human-specific neurological disorders are associated with mutations in this gene, suggest that *AUTS2* has an important role in the evolution of human cognitive traits.

## Materials and Methods

### Whole-mount *in situ* hybridization

Zebrafish embryos were collected from ABs or caspers [42] between 24 to 120 hpf and fixed in 4% paraformaldehyde buffered with 1× PBS (PFA). The zebrafish *auts2* (Open Biosystems EDR1052-4681254) cDNA clone was used to generate digoxygenin labeled probes. Whole-mount *in situ* hybridizations were performed according to standard protocols [43].

### Morpholino assays

Two morpholino (MO) antisense oligonucleotides targeting *auts2* were designed by Gene-Tools. One MO was designed to target the translational start site of *auts2* (GTGGAGAGTGTGTCAACACTAAAAT). The second was designed to target the splice junction between intron 2 and exon 3 of Ensembl Transcript ENSDART00000137928 (TCGACTACTGCTGTGAACAAAGAGA). A third 5 bp mismatch control for the translational MO (GTGGACACTGTGTGAAGACAAAAAT) was also designed. The MOs were diluted to 1 mM in deionized water and injected using standard techniques [44] into one cell-stage embryos. To rescue the morphant phenotypes, we transcribed full length human *AUTS2* RNA (Open Biosystems MHS1010-9204165) using the T7 message machine (Ambion) and co-injected it along with the translational MO at a concentration of 168 ng/ul. The HuC line was generously donated by Dr. Su Guo (UCSF). The Tg(mnx1∶GFP) (AB) line (formerly known as hb9) was obtained from the Zebrafish International Resource Center (ZIRC; http://zebrafish.org/zirc/home/guide.php). Fish where injected with MOs as described above and annotated using the Leica M165 FC microscope. At least 50 translational MO injected fish and controls were compared in all zebrafish lines used.

### Immunohistochemistry on zebrafish sections

AB zebrafish embryos injected with the *auts2* translational MO or the 5 bp control were fixed at 48 hpf in 4% PFA overnight at 4°C, then washed for 15 minutes at room temperature in PBS. Zebrafish were frozen into blocks using Tissue-Tek O.C.T. (Sakura Finetek) then sectioned (10–20 microns) using a Leica CM1850 cryostat and stained with Nissl (FD NeuroTechnologies). Morphant and control sections represent comparable planes. Staining with PCNA (DAKO, Monoclonal Mouse PCNA clone PC10) was done according to the manufacturer's protocol. Cell nuclei were visualized using DAPI (Invitrogen). Staining sections with TUNEL (Roche, *In Situ* Cell Death Detection Kit, TMR red) was done according to the manufacturer's protocol. Zebrafish sections were analyzed using the Leica M165 FC or the Nikon Eclipse E800 microscope. At least 25 fish were analyzed in each condition. Control and morphant pictures were taken with identical exposures and are representative of each condition. For TUNEL staining on sections, criteria for amount of cell death was based on the number of individual TUNEL positive cells identified in the midbrain and eye, indicative of cell death in those regions. For PCNA staining (cell cycle marker) on sections, criteria for amount of proliferation in the forebrain, midbrain and hindbrain was qualitatively evaluated due to the larger number of PCNA positive cells in morphants compared to controls.

### Zebrafish whole-mount immunohistochemistry

Casper zebrafish embryos injected with the *auts2* translational MO or the 5 bp control were fixed at 48 hpf overnight at 4°C in 4% PFA. For TUNEL staining, embryos were transferred to methanol for 30 minutes followed by rehydration in methanol/PBST (PBS with 0.1% tween). They were then placed in Proteinase K (10 µg/ml) for 5 minutes and postfixed in 4% PFA for 20 minutes. Embryos were later placed in prechilled ethanol∶acetic acid (2∶1) at −20°C for 10 minutes and then washed in PBST for 20 minutes followed by TUNEL staining using the *In Situ* Cell Death Detection Kit, TMR red (Roche) according to the manufacturer's protocol. Sensory neurons were analyzed using anti-HNK-1 (Sigma) followed by the goat anti-mouse IgM HRP secondary antibody (abcam, ab5930) using previously described methods [45]. HNK-1 positive cells where manually counted in 6 different control and morphant fish. Fish were analyzed using the Leica M165 FC or the Nikon Eclipse E800 microscope. At least 25 fish were analyzed in each condition. Control and morphant pictures were taken with identical exposures and are representative of each condition. For TUNEL whole mount staining, criteria for amount of cell death was based on the number of viewable individual TUNEL positive cells in the forebrain, midbrain and hindbrain. For HNK-1 staining, criteria for amount of sensory neurons was based on the number of individual HNK-1 positive cells counted in equal lengths of the trunk. Motor neuron axons were analyzed using anti-znp-1 (Developmental Studies Hybridoma Bank) followed by anti-mouse IgG HRP (GE Healthcare) using previously described methods [46].

### Transgenic enhancer assays

*AUTS2* enhancer candidate (AEC) sequences were selected based on evolutionary conservation (sequences showing ≥70% identity for at least 100 bp between human and chicken), E1A binding protein p300 (EP300) forebrain or hindbrain ChIP-Seq datasets [32], and nucleotide variants that define the human-Neanderthal sweep [17] (Table S1). PCR was carried out on human genomic DNA (Qiagen) using primers designed to amplify the AEC sequences (Table S1). Primers were designed such that they will have additional flanking sequences to the conserved, ChIP-Seq or human-Neanderthal accelerated sequences based on previous experiments that have shown this to be a reliable method for obtaining positive enhancer activity [47]. PCR products were cloned into the E1b-GFP-Tol2 enhancer assay vector containing an E1b minimal promoter followed by GFP [33]. They were then injected following standard procedures [46], [48] into at least 100 embryos per construct along with Tol2 mRNA [49], to facilitate genomic integration. GFP expression was observed and annotated up to 48 hpf. An enhancer was considered positive if at least 15% of all fish surviving to 48 hpf showed a consistent expression pattern after subtracting out percentages of tissue expression in fish injected with the empty enhancer vector. Notably, the empty vector showed particularly high background for heart and somitic muscle and as described all enhancer results were obtained after deducting its expression pattern. Thus, in order to call positive somitic muscle enhancer activity, over 26% (24hpf) or 40% (48hpf) of alive fish needed to show positive enhancer activity. To call a positive heart enhancer, 32% (24hpf) or 50% (48hpf) of alive fish needed show positive heart activity. For each construct, at least 50 fish were analyzed for GFP expression at 48 hpf. For the mouse enhancer assays, the same human genomic fragment used in zebrafish was transferred into a vector containing the *Hsp68* minimal promoter followed by a *LacZ* reporter gene [47], [50] and sequence verified to ensure the insert matched the human reference sequence. Sequences having rare variants were changed to the reference human genomic sequence by site-directed mutagenesis (Mutagenex or Quickchange II, Stratagene) and sequence verified for having the reference sequence. Transgenic mice were generated by Cyagen Biosciences using standard procedures [51]. Embryos were harvested at E12.5 and stained for LacZ expression using standard procedures [47]. Mouse embryos selected for sectioning were placed in an overnight cryoprotection stage using 30% sucrose in PBS. Mice were frozen into blocks using Tissue-Tek O.C.T. (Sakura Finetek) then sectioned (20 microns) using a Leica CM1850 cryostat and stained with Nuclear Fast Red Solution (Sigma-Aldrich) for one minute. There is no human subjects work involved in this article. All animal work was approved by the UCSF Institutional Animal Care and Use Committee (protocol number AN084690).

## Supporting Information

Figure S1 *auts2* expression and morphant phenotype. (A) Whole-mount *in situ* hybridization of *auts2* shows that it is expressed in the forebrain (fb) (including olfactory organs), midbrain (mb), hindbrain (hb), spinal cord (sc), the caudal peduncle and eye at 24hpf. At 48hpf, *auts2* is expressed in the brain, pectoral fin and eye. At 120 hpf expression is restricted to the brain, primarily the midbrain, and weakly in the eye. (B) Fish injected with the 5 bp translational MO mismatch control have indistinguishable morphology as wild type fish at 24, 48 and 120 hpf. (C) Injection of the *auts2* translational MO results in fish with a stunted development phenotype that includes smaller heads, eyes, bodies and fins. (E) Injection of the *auts2* splice-blocking MO shows a similar but less severe phenotype than the *auts2* translational MO. (E) The *auts2* translational MO phenotype is partially rescued by co-injecting the full length human *AUTS2*. Note the longer body and larger brain compared to the translational and splicing morphant fish. MO injected fish in C, D, and E are scaled to the 5 bp injected control fish in B.
(TIF)

Figure S2 Histological phenotype of *auts2* morphants (A) Nissl staining shows a reduction in neuron territory, primarily in the midbrain, of fish injected with the translational MO compared to 5 bp mismatch controls at 48 hpf. (B) TUNEL stained sections show fewer apoptotic cells in the optic tectum (white arrowhead) and the retina (green arrowhead) in 48 hpf *auts2* morphants versus the 5 bp translational MO mismatch control. (C–E) Coronal sections stained with PCNA, DAPI and overlays show an increase in cell proliferation in the translational morphant fish compared to the 5 bp mismatch control in the mesencephalon, diencephalon and retina.
(TIF)

Figure S3 znp-1 antibody on control and morphant fish. The motor neurons axons of the morphant fish are different than the controls, signified by a drastic increase in the amount of branching (red arrow).
(TIF)

Figure S4 *AUTS2* enhancer candidates (AECs) positive for enhancer activity in zebrafish. A representative fish of each positive AEC enhancer is shown. The number in the top right of every image is the AEC number and the hours post fertilization (hpf) when the picture was taken is indicated in the bottom right. Their tissue-specific expression pattern is denoted in Table S1 and http://zen.ucsf.edu.
(TIF)

Figure S5 The enhancer expression patterns of E12.5 LacZ positive mouse embryos injected with AEC10, 24, 29 and 32. 12 out of 13 AEC10 E12.5 mouse embryos show midbrain enhancer expression and 12 out of 13 have eye expression. 4 out of 5 AEC24 E12.5 mouse embryos show eye enhancer expression. 4 out of 6 AEC29 E12.5 embryos show olfactory epithelium enhancer expression and 6 out of 6 have eye expression. 4 out of 4 AEC32 E12.5 embryos show midbrain, forebrain, hindbrain and eye enhancer expression. Additional mouse embryos for enhancers AEC12, 21 and 27 can be found online at the VISTA enhancer browser website [34] (http://enhancer.lbl.gov/) as hs1660, hs1425 and hs658, respectively.
(TIF)

Table S1 *AUTS2* enhancer candidates (AECs) selected for enhancer assays.
(XLSX)

Video S1 *auts2* 5 bp MO control injected zebrafish show normal response when prodded with a pipette tip at 48 hpf.
(AVI)

Video S2 *auts2* splicing MO injected zebrafish, that have a less severe morphological phenotype than the translational morphants, show decreased movement when prodded with a pipette tip at 48 hpf.
(AVI)
