# Supplementary figures and images for: Function and Regulation of AUTS2, a Gene Implicated in Autism and Human Evolution
Source: PLoS Genet. 2013 Jan 17;9(1):e1003221. doi: 10.1371/journal.pgen.1003221 (PMC3547868; doi:10.1371/journal.pgen.1003221)

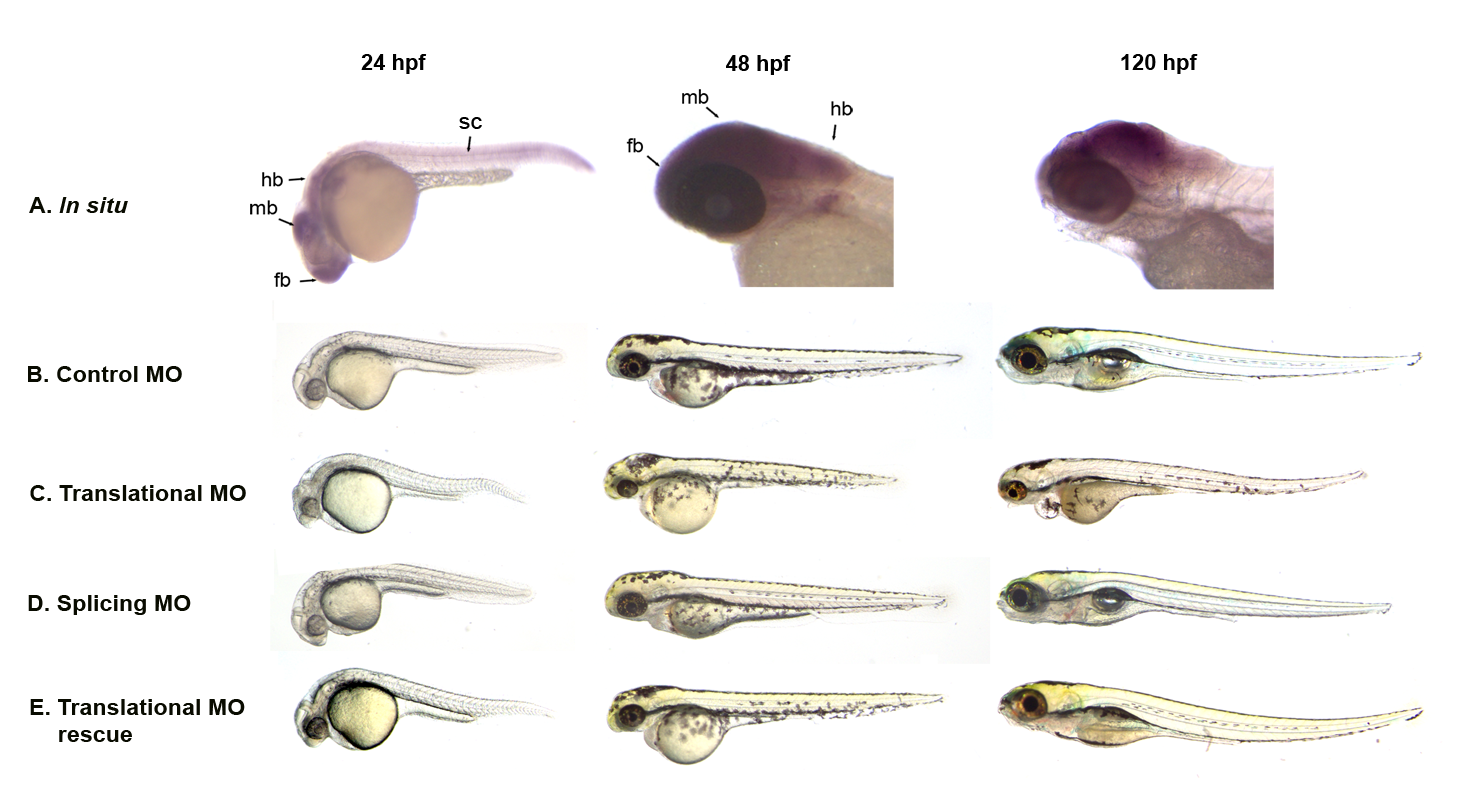

Supplement: Figure S1 — auts2 expression and morphant phenotype. (A) Whole-mount in situ hybridization of auts2 shows that it is expressed in the forebrain (fb) (including olfactory organs), midbrain (mb), hindbrain (hb), spinal cord (sc), the caudal peduncle and eye at 24hpf. At 48hpf, auts2 is expressed in the brain, pectoral fin and eye. At 120 hpf expression is restricted to the brain, primarily the midbrain, and weakly in the eye. (B) Fish injected with the 5 bp translational MO mismatch control have indistinguishable morphology as wild type fish at 24, 48 and 120 hpf. (C) Injection of the auts2 translational MO results in fish with a stunted development phenotype that includes smaller heads, eyes, bodies and fins. (E) Injection of the auts2 splice-blocking MO shows a similar but less severe phenotype than the auts2 translational MO. (E) The auts2 translational MO phenotype is partially rescued by co-injecting the full length human AUTS2. Note the longer body and larger brain compared to the translational and splicing morphant fish. MO injected fish in C, D, and E are scaled to the 5 bp injected control fish in B. (TIF) [file pgen.1003221.s001.tif]

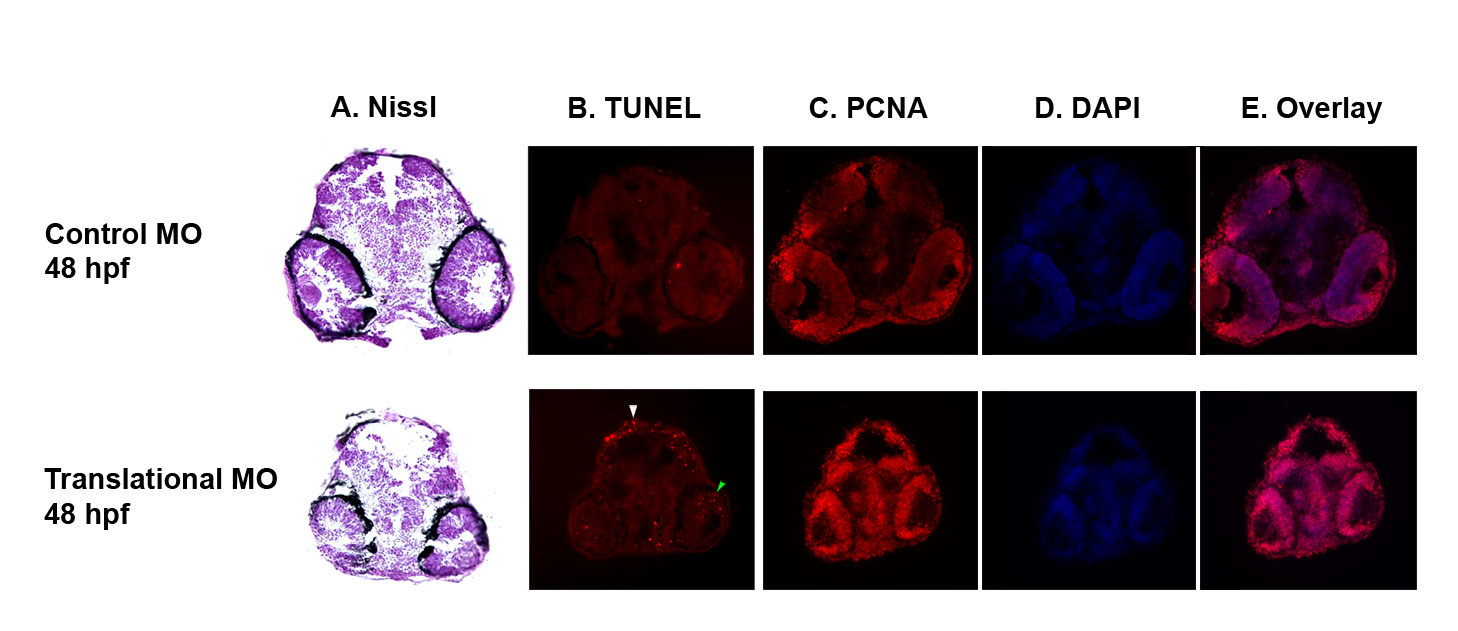

Supplement: Figure S2 — Histological phenotype of auts2 morphants (A) Nissl staining shows a reduction in neuron territory, primarily in the midbrain, of fish injected with the translational MO compared to 5 bp mismatch controls at 48 hpf. (B) TUNEL stained sections show fewer apoptotic cells in the optic tectum (white arrowhead) and the retina (green arrowhead) in 48 hpf auts2 morphants versus the 5 bp translational MO mismatch control. (C–E) Coronal sections stained with PCNA, DAPI and overlays show an increase in cell proliferation in the translational morphant fish compared to the 5 bp mismatch control in the mesencephalon, diencephalon and retina. (TIF) [file pgen.1003221.s002.tif]

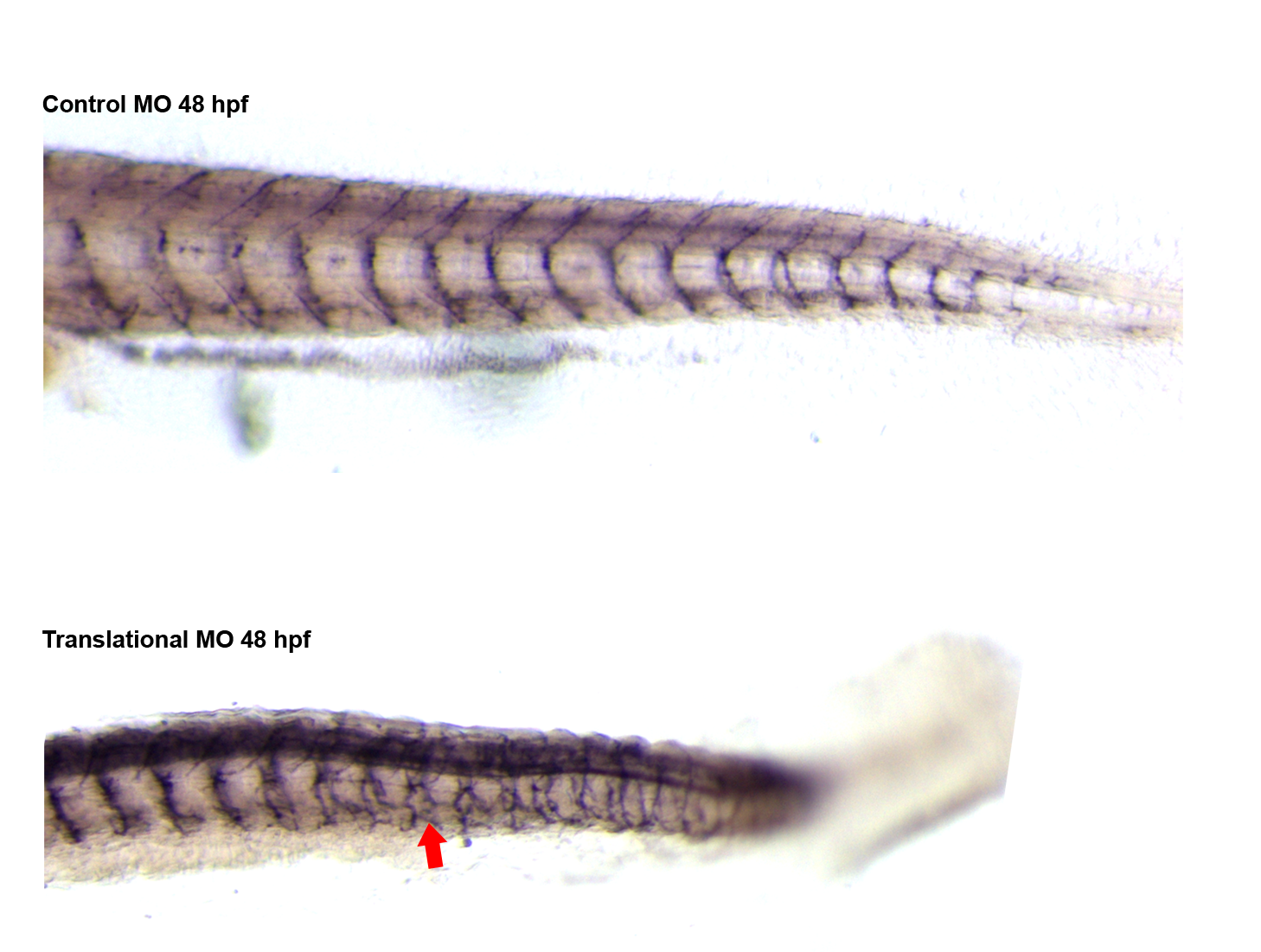

Supplement: Figure S3 — znp-1 antibody on control and morphant fish. The motor neurons axons of the morphant fish are different than the controls, signified by a drastic increase in the amount of branching (red arrow). (TIF) [file pgen.1003221.s003.tif]

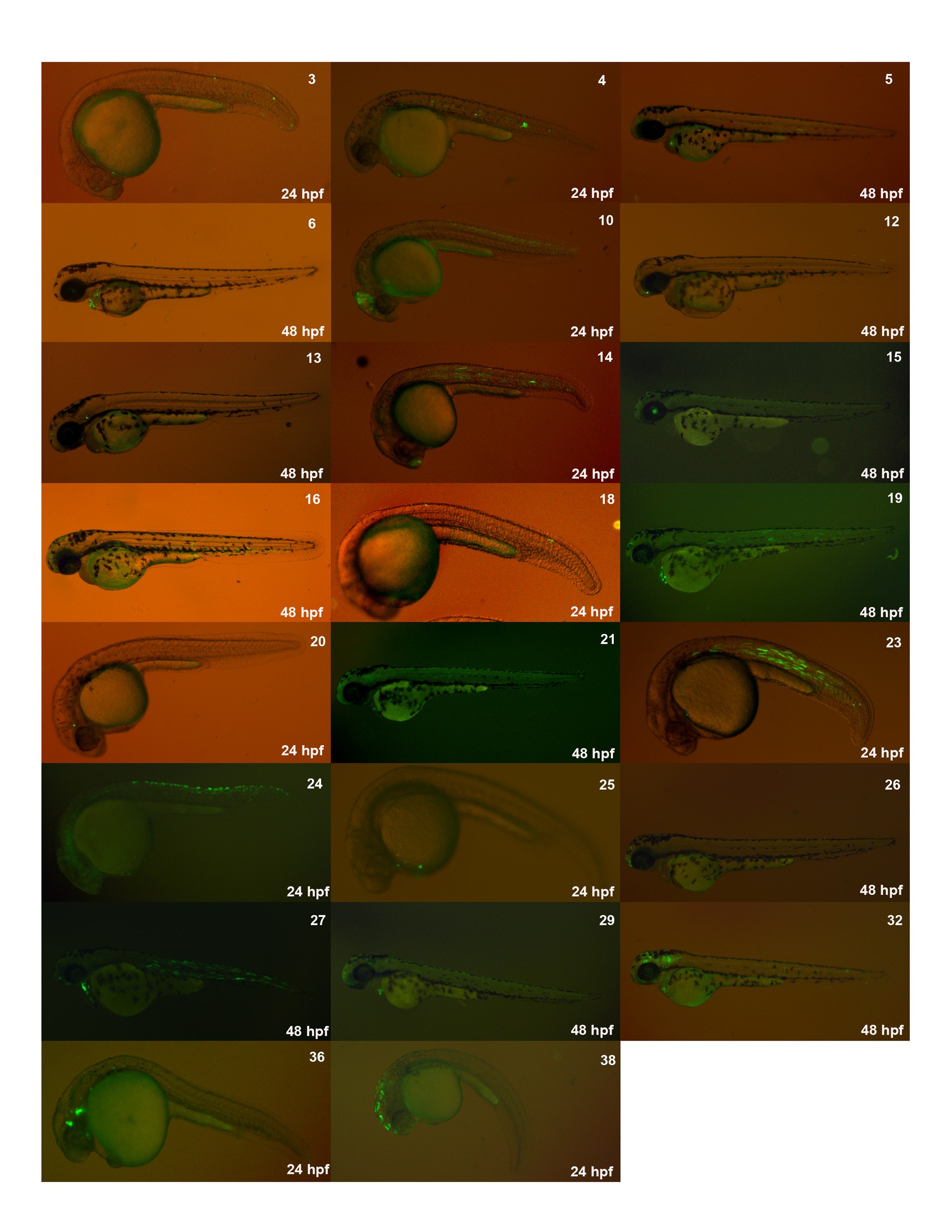

Supplement: Figure S4 — AUTS2 enhancer candidates (AECs) positive for enhancer activity in zebrafish. A representative fish of each positive AEC enhancer is shown. The number in the top right of every image is the AEC number and the hours post fertilization (hpf) when the picture was taken is indicated in the bottom right. Their tissue-specific expression pattern is denoted in Table S1 and http://zen.ucsf.edu. (TIF) [file pgen.1003221.s004.tif]

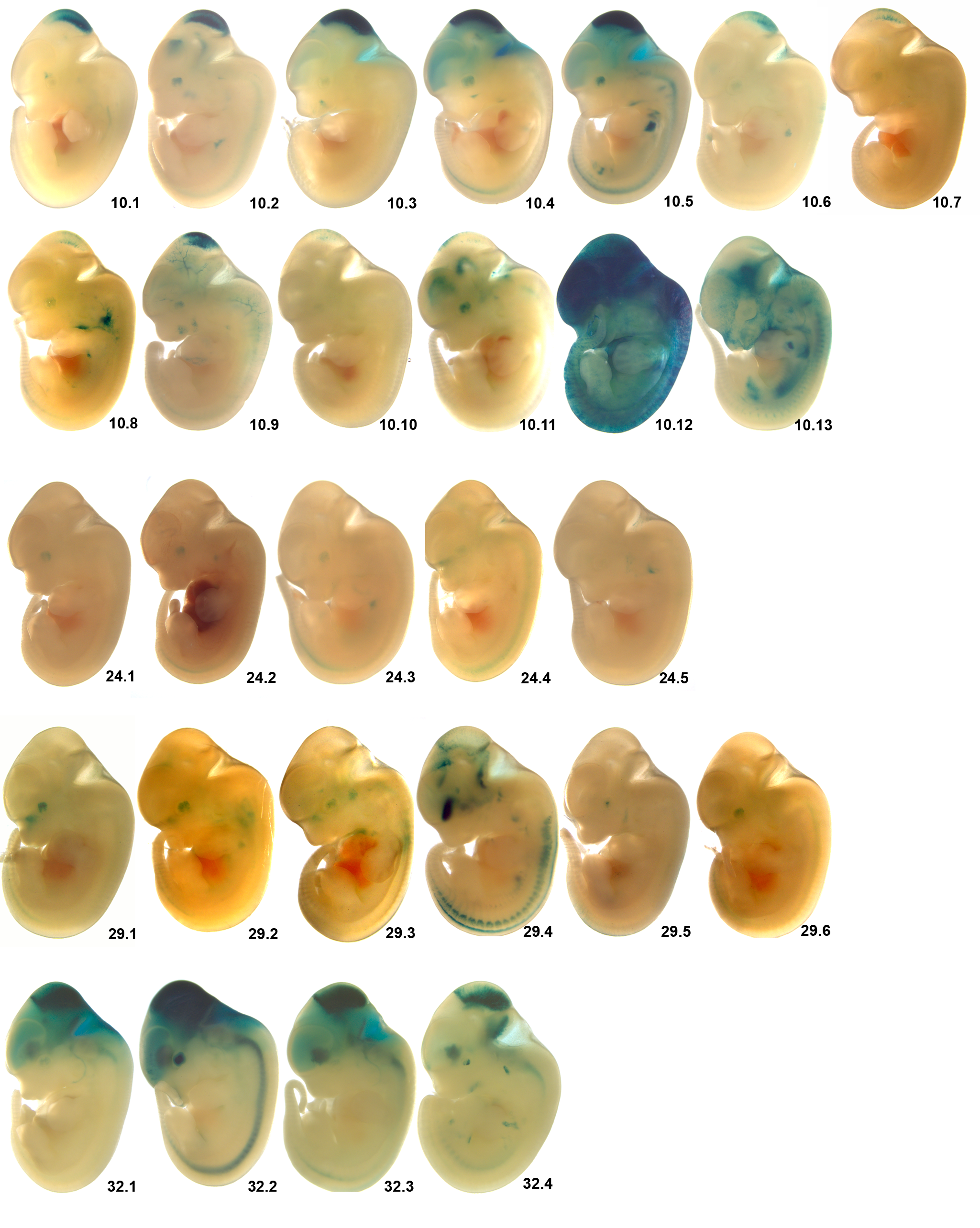

Supplement: Figure S5 — The enhancer expression patterns of E12.5 LacZ positive mouse embryos injected with AEC10, 24, 29 and 32. 12 out of 13 AEC10 E12.5 mouse embryos show midbrain enhancer expression and 12 out of 13 have eye expression. 4 out of 5 AEC24 E12.5 mouse embryos show eye enhancer expression. 4 out of 6 AEC29 E12.5 embryos show olfactory epithelium enhancer expression and 6 out of 6 have eye expression. 4 out of 4 AEC32 E12.5 embryos show midbrain, forebrain, hindbrain and eye enhancer expression. Additional mouse embryos for enhancers AEC12, 21 and 27 can be found online at the VISTA enhancer browser website [34] (http://enhancer.lbl.gov/) as hs1660, hs1425 and hs658, respectively. (TIF) [file pgen.1003221.s005.tif]
